# Diversity of Expression Types of *Ht* Genes Conferring Resistance in Maize to *Exserohilum turcicum*

**DOI:** 10.3389/fpls.2020.607850

**Published:** 2020-12-17

**Authors:** Barbara Ludwig Navarro, Hendrik Hanekamp, Birger Koopmann, Andreas von Tiedemann

**Affiliations:** ^1^Division of Plant Pathology and Crop Protection, Department of Crop Sciences, Georg-August-Universität Göttingen, Göttingen, Germany; ^2^Plant Protection Office, Chamber of Agriculture Lower Saxony, Hannover, Germany

**Keywords:** R genes, *Setosphaeria turcica*, northern corn leaf blight, qualitative resistance, histology

## Abstract

Northern corn leaf blight (NCLB) is an important leaf disease in maize (*Zea* mays) worldwide and is spreading into new areas with expanding maize cultivation, like Germany. *Exserohilum turcicum*, causal agent of NCLB, infects and colonizes leaf tissue and induces elongated necrotic lesions. Disease control is based on fungicide application and resistant cultivars displaying monogenic resistance. Symptom expression and resistance mechanisms differ in plants carrying different resistance genes. Therefore, histological studies and DNA quantification were performed to compare the pathogenesis of *E. turcicum* races in maize lines exhibiting compatible or incompatible interactions. Maize plants from the differential line B37 with and without resistance genes *Ht1, Ht2, Ht3*, and *Htn1* were inoculated with either incompatible or compatible races (race 0, race 1 and race 23N) of *E. turcicum*. Leaf segments from healthy and inoculated plants were collected at five different stages of infection and disease development from penetration (0–1 days post inoculation - dpi), until full symptom expression (14–18 dpi). Symptoms of resistance responses conveyed by the different *Ht* genes considerably differed between *Ht1* (necrotic lesions with chlorosis), *Ht2* (chlorosis and small lesions), *Ht3* (chlorotic spots) and *Htn1* (no lesions or wilt-type lesions). In incompatible interactions, fungal DNA was only detected in very low amounts. At 10 dpi, DNA content was elevated in all compatible interactions. Histological studies with Chlorazol Black E staining indicated that *E. turcicum* formed appressoria and penetrated the leaf surface directly in both types of interaction. In contrast to incompatible interactions, however, the pathogen was able to penetrate into xylem vessels at 6 dpi in compatible interactions and strongly colonized the mesophyll at 12 dpi, which is considered the crucial process differentiating susceptible from resistant interactions. Following the distinct symptom expressions, resistance mechanisms conferred by *Ht1, Ht2, Ht3*, and *Htn1* genes apparently are different. Lower disease levels and a delayed progress of infection in compatible interactions with resistant lines imply that maize R genes to *E. turcicum* are associated with or confer additional quantitative resistance.

## Introduction

Northern corn leaf blight (NCLB) caused by the ascomycete *Exserohilum turcicum* [(Pass.) Leonard and Suggs], synonym *Setosphaeria turcica* [(Luttrel) Leonard and Suggs] has spread worldwide into regions where maize is cultivated. Yield losses up to 44% were recorded in susceptible hybrids at high disease severity levels between 52 and 100% during the full dent stage (Bowen and Paxton, 1988). Yield losses depend on the level of host resistance, disease severity, plant phenological growth stage during infection, and position of the infected leaves (Levy and Pataky, 1992). Two to 3 weeks after pollination, high levels of disease severity caused yield losses between 40 and 70% (Levy and Pataky, 1992). In addition, high disease severity of the leaf at the ear node is correlated with high yield losses (Levy and Leonard, 1990).

The pathogen can survive as chlamydospore in plant debris (Levy, 1995) and inoculum can be spread by rain and wind (Galiano-Carneiro and Miedaner, 2017). Under conditions of high humidity, conidia are able to germinate after one-hour in a broad temperature range (20–30°C) (Levy and Cohen, 1983). Conidia germination is bipolar and an appressorium is usually formed at the end of germ tubes (Jennings and Ullstrup, 1957). Appressoria formation starts about 3 h after inoculation (Levy and Cohen, 1983). Infection by *E. turcicum* is usually initiated by direct penetration through the cuticle and epidermis. Penetration through stomata has been observed at 10% of penetration sites (Hilu and Hooker, 1964). As a hemibiotroph, after penetration of the epidermis, hyphae invaginate the membrane in the first stages of infection and a spherical intracytoplasmic vesicle is formed (Hilu and Hooker, 1964; Knox-Davies, 1974). After the primary stage of infection, hyphae start colonization of adjacent cells in the mesophyll (Knox-Davies, 1974) until xylem vessels are reached (Muiru et al., 2008; Kotze et al., 2019). In later stages of infection, the pathogen may leave the xylem, colonize mesophyll cells, and form conidiophores on the leaf surface, which will disperse the conidia (Kotze et al., 2019). The sexual stage was first reported in fields in Thailand. Sexual reproduction only occurs in populations with both mating types. Moreover, perithecia induction and maturation requires specific climatic conditions (Bunkoed et al., 2014).

Typical symptoms of NCLB are gray-green elongated necrotic lesions (Galiano-Carneiro and Miedaner, 2017). Disease levels may range from small lesions to necrosis of whole leaves (Welz and Geiger, 2000). Seedlings are more susceptible to disease than young plants (Levy and Cohen, 1983). Fungicide application and host resistance are typically applied for NCLB control (Galiano-Carneiro and Miedaner, 2017). However, resistant cultivars are more frequently used in maize fields worldwide. Host resistance is based on qualitative and/or quantitative resistance. In breeding programs, qualitative resistance can be a faster strategy to improve resistance on new hybrids (Galiano-Carneiro and Miedaner, 2017). Resistance mechanisms and, consequently, phenotypes might differ in plants bearing different resistance genes. The resistance phenotype typically expressed by the resistance genes *Ht1*, *Ht2*, and *Ht3* is a chlorosis while the resistance mechanism described for plants harboring *Htn1* is an extended latent period (Levy and Pataky, 1992).

*Ht1* was first found in two lines, “GE440,” from the United States, and “Ladyfinger,” a popcorn variety from Peru (Hooker, 1963). The reaction on hybrids bearing this resistance gene are characterized by chlorotic lesions, a delay in necrosis, and inhibition of fungal sporulation. *Ht2* was discovered in a line from Australia, “NN14B,” which displayed chlorotic lesions (Hooker, 1977). In the first description of the *Ht2* gene, lower resistance levels were mentioned when compared to the *Ht1* gene. The third R gene (*Ht3*) described for *E. turcicum* also expressed resistance by chlorotic lesions and was introgressed from a grass, *Tripsacum floridanum*, native to Cuba and Florida (Hooker, 1981). Another resistance gene used in breeding programs is known as *Htn1* and is derived from the Mexican maize variety “Pepitilla” and the resistance mechanism described is a delay in infection (Gevers, 1975).

The introduction of qualitative resistance in commercial hybrids may promote the selection of new physiological races. The race nomenclature for *E. turcicum* in maize is based on the resistance gene(s) which the isolate can overcome (Leonard et al., 1989). Race 0 only infects plants without any resistance genes. Conversely, race 23N isolates are virulent on plants carrying resistance genes *Ht2*, *Ht3*, and *Htn1*. Several race monitoring studies using *E. turcicum* populations from different regions of the world have identified races that have overcome all major resistance genes. In the United States, the frequency of isolates virulent on maize lines containing *Ht1* was higher than the frequency of race 0 isolates due to widespread cultivation of commercial hybrids with the *Ht1* resistance gene in recent years (Ferguson and Carson, 2007; Weems and Bradley, 2018).

*Ht* resistance genes have been widely used in breeding programs (Welz and Geiger, 2000; Galiano-Carneiro and Miedaner, 2017). It has been hypothesized that fungal colonization, especially xylem penetration may differ between compatible and incompatible interactions. In an incompatible interaction, hyphae are restricted to xylem vessels (Muiru et al., 2008; Kotze et al., 2019). Therefore, the aim of this work was to characterize and quantify fungal colonization in plants carrying the resistance genes *Ht1, Ht2, Ht3*, and *Htn1* with isolates displaying compatible and incompatible interactions. The *in situ* characterization of fungal growth in host tissue was based on five different time points from initial penetration through symptom differentiation between interactions. Fungal DNA quantification and histological studies were performed with the differential set of near isogenic inbred lines of the recurrent parent B37 without resistance genes and near isogenic lines harboring different *Ht* resistance genes.

## Materials and Methods

### Plant Material, Fungal Strains and Inoculation

Maize plants from the differential set based on near isogenic inbred lines of the recurrent parent B37 with no qualitative resistance gene and with resistance genes *Ht1, Ht2, Ht3*, and *Htn1* were cultivated in the greenhouse at 24 ± 3°C, 70% relative humidity, a day/night light regime of 14/10 h and light intensity of 120 ± 10 μmol m^–2^ s^–1^. Two seeds per pot (11 cm × 11 cm × 10 cm) were sown in a mixture of compost, clay, and sand in the proportion 3:3:1. Seeds were provided from KWS Saat SE (Einbeck, Germany). Maize plants were inoculated using a sprayer when the fifth and sixth leaves were unfolded, about 30 days after sowing. Incompatible interactions were induced by inoculating race 0 on near isogenic lines B37*Ht1*, B37*Ht2*, B37*Ht*3, and B37*Htn1*, whereas the compatible interaction was studied by inoculating the same race 0 isolate on B37 without resistance genes. Furthermore, compatible interactions were analyzed by inoculating race 1 on B37*Ht1*, and race 23N on B37*Ht2*, B37*Ht3*, and B37*Htn1* lines (Supplementary Table 1). The origin of isolates and race determination were described previously (Hanekamp, 2016). Each plant received seven ml of a conidia suspension at a concentration of 3,000 conidia ml^–1^ and was maintained in a humidity chamber for 24 h.

Fourteen days post inoculation (dpi), four plants per treatment were evaluated to confirm compatible and incompatible interactions between the differential lines and isolates (Supplementary Table 1). Leaf samples were collected from the inoculated area with visual symptoms. Disease phenotyping, disease rating, fungal DNA quantification, and microscopic studies on fungal colonization were based on five different time points: penetration (0–1 days post inoculation, dpi), first stages of infection (2–4 dpi), pre-symptomatic disease stage (5–7 dpi), first symptom expression (10–12 dpi), and occurrence of symptoms distinguished between interactions (14–18 dpi). Before sampling at the last time point, disease severity was evaluated in ten plants based on a diagrammatic scale (Pataky, 1992). Disease severity of the replicated experiment is presented in Supplementary Figure 1.

### DNA Quantification of *Exserohilum turcicum* in Infected Leaves

Leaf samples for DNA quantification were harvested right after inoculation (0 dpi), and three, six, ten, and fourteen-days post inoculation (dpi). Nine plants were harvested per treatment and timepoint. The fourth and fifth leaves from three plants were harvested and pooled together in one biological replicate. Three biological replicates were used per treatment and timepoint. For the DNA standard curve, the race 0 isolate was grown in liquid Czapek Dox Medium at 22°C in the dark. The mycelial culture was shaken at 100 rpm for 14 days and then filtered by vacuum suction. The mycelium was frozen, lyophilized, ground, and homogenized with a mixer mill (Retsch^®^ MM400, Haan, Germany). Genomic DNA (gDNA) extraction was performed with the CTAB method (Brandfass and Karlovsky, 2008), where 1 ml of CTAB-buffer (20 mM Na-EDTA, 0.13 M sorbitol, 30 mM N-laurylsarcosine, 20 mM CTAB, 0.8 M NaCl, 10 mM Tris – pH 8.0 adjusted with NaOH) were added to 50 mg ground leaf sample. Proteinase K (1 μl from 20 mg ml^–1^ stock solution) was added to each sample. The mixture was treated in an ultrasonic bath for 5 s then incubated for 10 min at 42°C and 10 min at 65°C (tubes were shaken during incubation). After incubation, 800 μl of chloroform-isoamyl alcohol (24:1) were added and tubes were shaken. Samples were incubated for 10 min on ice, then centrifuged at 13,000 × *g* for 10 min (Hettich Zentrifugen Mikro 220R, Germany). The supernatant was transferred to another tube with 200 μl of 30% (w/v) PEG and 100 μl of 5 M NaCl. The pellet was washed with 70% (v/v) ethanol, then dried at room temperature. The dry pellet was dissolved in 100 μl TE buffer pH 8.0 (0.1 M Tris, EDTA 10 mM) and stored at −20°C. After DNA extraction, 1 μl of each sample was placed in an agarose gel (1%) and electrophoresis was performed to verify the DNA extraction procedure.

The amount of DNA was measured by electrophoresis in an agarose gel (1%) and compared with known DNA concentrations of bacteriophage Lambda. Samples with a high genomic DNA (gDNA) concentration were diluted 1:10. The dilution factor was considered in further calculations. A standard curve was obtained by diluting fungal DNA from 1,000 to 0.01 pg × μl^–1^ (1,000, 100, 10, 1, 0.1, and 0.01 pg × μl^–1^) to quantify the target sequence by qPCR. The calibration curve was based on a linear regression of the quantification cycle value versus the logarithmic values of known gDNA. Data were analyzed with the software BioRad CFX Maestro 1.1 (Fa. Bio-Rad).

Quantitative polymerase chain reaction (qPCR) analysis was performed with a primer pair designed to amplify the pathogen specific internal transcribed spacer (ITS) region (Beck, 1998). The primer pair used was (forward) JB 586 (5′-TGGCAATCAGTGCTCTGCTG-3′) and (reverse) JB 595 (5′-TCCGAGGTCAAAATGTGAGAG-3′), resulting in an amplicon size of 485 base pairs. PCR reactions were performed with 5 μl of the premix qPCR BIO SyGreen Mix Lo-ROX (PCR Biosystems, London, United Kingdom) with a primer concentration of 0.4 μM and 1 μl from the DNA sample. The final volume of the reaction was 10 μl. The optimal thermal cycling conditions (CFX384 Thermocycler - Biorad, Rüdigheim, Germany) were 94°C for 3 min, followed by 40 cycles of 94°C for 5 s (denaturation), 63.5°C for 15 s (annealing), 72°C for 15 s (elongation), and 72°C for 5 min for final elongation. Three technical replicates were performed for each biological replicate.

Fungal DNA contents were compared between lines in the compatible interaction within every timepoint, for 10 dpi and 14 dpi by analysis of variance (ANOVA) and multiple comparison applying *post hoc* Tukey test (*p* ≤ 0.05) performed in the R software 3.6.0 (R Core Team, 2020) and graphics were generated in the software Microsoft Excel 2016. Data of the replicated experiment is presented in Supplementary Figure 2.

### Histological Studies

Leaf segments for the histological studies were collected at 1, 3, 6, 12, and 18 dpi. The fourth and fifth leaves from two plants were collected, resulting in four biological replicates per treatment and sampling time point. For every sampled leaf, six square centimeter (2 × 3 cm^2^) leaf segments were cut and fixed in FAA-solution (90 ml of ethanol 70%, 5 ml formaldehyde 36%, and 5 ml acetic acid 99%) and stored at room temperature. Leaf pigments were removed in two subsequent steps. First, they were incubated in 70% ethanol for 2 h at room temperature and then washed with water. This step was followed by incubation in a water bath at 90°C for 2.5 h in closed flask containing 2 M potassium hydroxide (KOH) in a 90°C water bath for 2.5 h. After bleaching, samples were washed with tap water and stained with Chlorazol Black E (CBE) (Sigma Aldrich) solution [0.03% (w/v) chlorazol black E; lactic acid, glycerin, distilled water in the proportion 1:1:1] at 60°C in a water bath overnight (adapted from Wilcox and Marsh, 1964). After staining with CBE, leaf segments were transferred in 50% glycerin and analyzed with light microscopy within the next 48 h.

**FIGURE 1:**
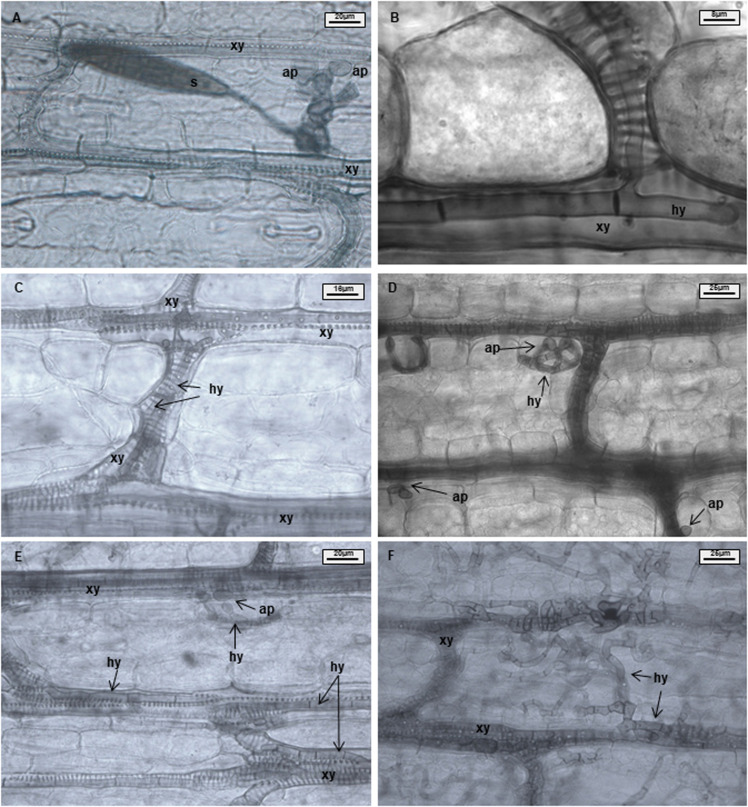
Maize leaves infected with *Exserohilum turcicum* after staining with Chlorazol Black E (CBE). Light microscopical images of the mesophyll. Appressorium-like structures formed from hyphae in the mesophyll; hyphae originating from a germinated spore penetrate the epidermis and colonize the mesophyll **(A)**. One hypha colonizing a xylem vessel **(B)**. Hyphae growing in a xylem vessel **(C)**. Mesophyll penetration/colonization takes place at a different location than initial penetration **(D)**. Appressorium-like structure formed from hyphae inside a xylem vessel **(E)**. Xylem vessels and mesophyll colonized by the fungus **(F)**. B37*Ht2* incompatible interaction at 6 days post inoculation (dpi) **(A)**, B37 at 6 dpi **(B,C,E)**, B37 at 12 dpi **(D)**, and B37 at 18 dpi **(F)**. Hyphae (hy), xylem (xy), appressoria (ap), and spore (s).

Samples were analyzed using 50% glycerin as mounting fluid. Three parameters were evaluated during light microscopy analysis: xylem penetration efficiency (XPE), xylem colonization efficiency (XCE), and mesophyll colonization efficiency (MCE). Effective xylem penetration was considered when hyphae were able to penetrate the xylem vessel (Figures 1A,B). Xylem colonization occurred when two or more hyphae were visible inside the xylem vessel (Figure 1C). Mesophyll colonization was considered when hyphae left the xylem and colonized mesophyll tissue in a region different from the penetration site (Figures 1D–F). Ten penetration sites were evaluated per sample, resulting in forty penetration sites being studied per treatment and time point. XPE, XCE, and MCE were calculated by dividing the number of successful penetrations or colonizations to the number of evaluated penetration sites and transformed to percentage. Data were analyzed with the software Microsoft Excel 2016 and Statistica 13.0 (Statsoft, Tulsa, OK, United States). Data from each resistant line were compared with B37 using a Chi-square test (^∗^*p* ≤ 0.05, ^∗∗^*p* ≤ 0.01, and ^∗∗∗^*p* ≤ 0.001). Fungal colonization was illustrated using the Corel Draw graphics suite X8 software (Corel Corporation, Ottawa, ON, Canada).

## Results

### Symptomology

**FIGURE 2:**
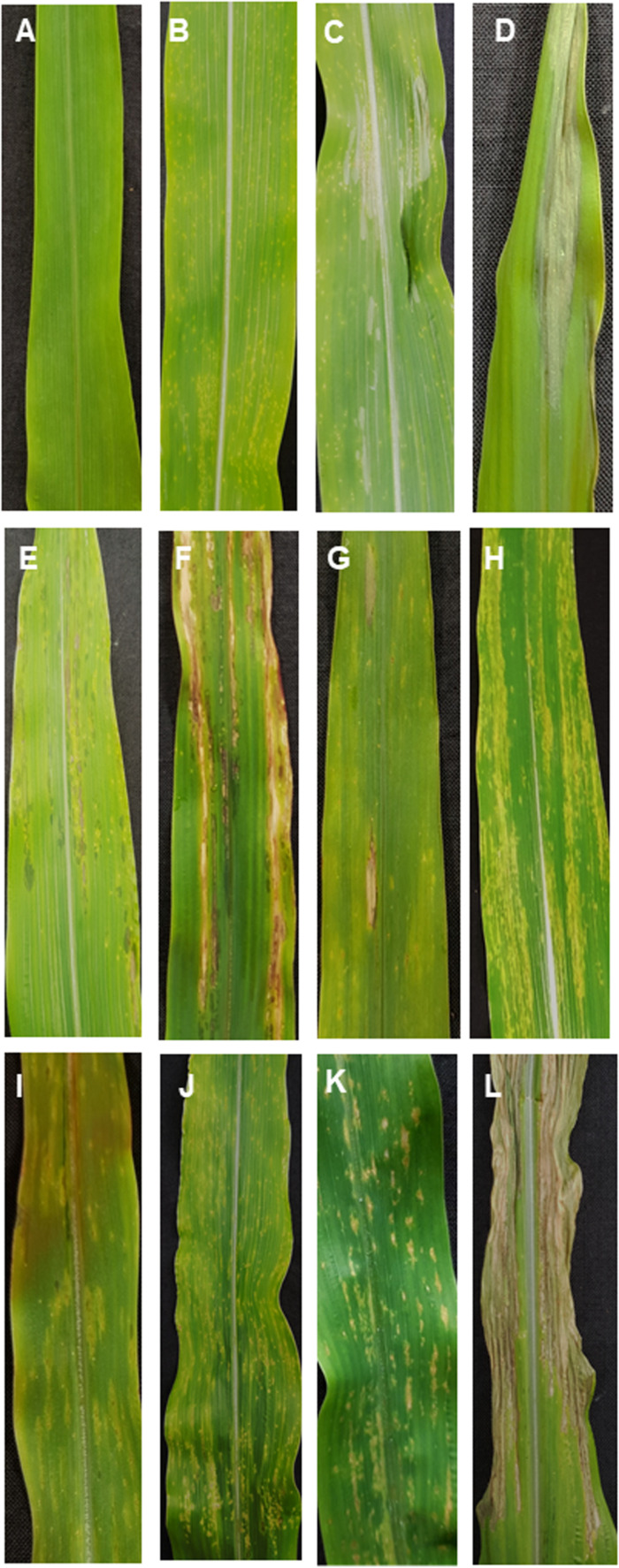
Symptomology of *Exserohilum turcicum* leaf infection on maize differential lines B37 without resistance genes and with the resistance genes *Ht1, Ht2, Ht3*, and *Htn1*. Symptom development in a compatible interaction (race 1 or race 23N) one-day post inoculation (dpi) **(A)**, 3 dpi **(B)**, 10 dpi **(C)**, and 14 dpi **(D)**. In the incompatible interaction (race 0 isolate), B37*Ht1* small soaked lesions are present at 6 dpi **(E)** and strong necrosis surrounded by chlorosis occurs at 14 dpi **(F)**. Symptoms of an incompatible interaction at 14 dpi on B37*Ht2* **(G,H)**, B37*Ht3* **(I)**, and B37*Htn1* **(J)**. B37*Htn1* plants display yellow spots on older leaves **(K)**. Strong necrosis with brownish lesions was observed in the compatible interaction with B37*Htn1* **(L)**.

Disease symptoms in incompatible interactions were mostly characterized by chlorosis, while chlorosis was absent in compatible interactions. In the compatible interaction, symptoms of necrosis developed and were characterized by strong leaf blight. At 1 dpi, most plants showed no symptoms (Figure 2A). It was possible to observe slightly water-soaked spots on some leaves when illuminated from the backside. The first chlorotic spots were found in both interactions at 3 dpi (Figure 2B). Six-days post inoculation, all plants and interactions still presented chlorotic spots, except for the incompatible interaction B37*Ht1*, where yellow spots had developed into elongated soaked lesions (Figure 2E). Ten-days post inoculation, the first gray necrotic lesions were observed in compatible interactions (Figure 2C). Differences in symptoms between compatible and incompatible interactions were clearly distinguishable in almost all plants at 14 dpi (Figures 2D,F–J).

**FIGURE 3:**
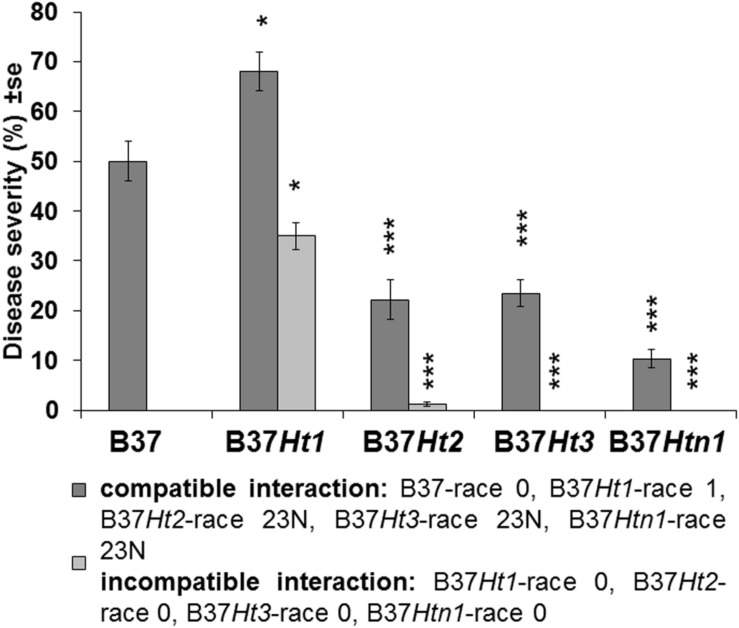
Disease severity and standard error for compatible and incompatible interactions between *Exserohilum turcicum* and the maize lines B37 without resistance genes and with resistance genes *Ht1, Ht2, Ht3*, and *Htn1*. Disease severity was evaluated 14 days post inoculation (dpi). Ten plants were evaluated per treatment (*n* = 10 plants). Data from the first replication experiment are presented in the graph. Data from each line with resistance genes was compared with B37 by Mann–Whitney-*U* test (*p ≤ 0.05, **p ≤ 0.01, and ***p ≤ 0.001).

Fourteen days post inoculation, B37 presented typical gray necrotic lesions (Figure 2D) and sometimes the leaf was completely dried (Figure 2L). In the incompatible interaction, each *Ht-*resistance gene expressed different symptoms of resistance indicating differences in the underlying resistance mechanisms. B37*Ht1* presented chlorosis with strong necrosis and developed a completely dry leaf (Figure 2F). In B37*Ht2*, a distinction between compatible and incompatible interaction based on chlorosis and necrosis was not clear (Figures 2G,H). Some plants expressed chlorosis, while others expressed small gray lesions, even in inoculations with the same isolate and in the same experiment. In contrast, symptom expression by B37*Ht*3 was quite uniform compared to the other resistant lines. B37*Ht*3 consistently formed yellow spots at penetration sites (Figure 2I). Older leaves from B37*Htn1* developed small wilt-type spots, independent of the kind of inoculation (Figure 2K). Furthermore, the disease levels in the compatible interactions varied between B37 and differential lines with resistance genes. B37*Ht1* showed higher disease severity than B37, with an average of 63 and 43%, respectively. Lines B37*Ht2*, B37*Ht3*, and B37*Htn1* displayed fewer symptoms than B37. In the incompatible interaction, B37*Ht3* and B37*Htn1* did not develop any necrosis, in contrast to B37*Ht1* which showed strong necrosis, resulting in an average disease severity of 42%. In the incompatible interaction, B37*Ht2* some leaves showed small gray lesions but disease severity was lower than 5% (Figure 3).

### DNA Quantification of *Exserohilum turcicum*

**FIGURE 4:**
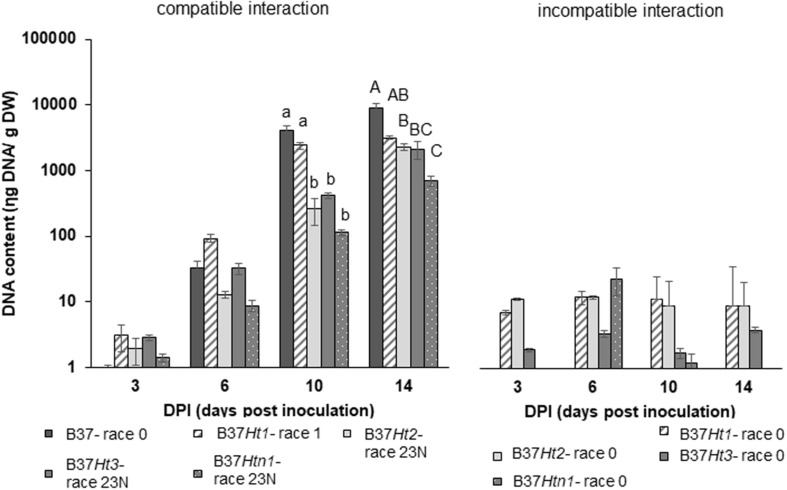
DNA-contents with standard errors for compatible and incompatible interactions between *Exserohilum turcicum* and the maize lines B37 without resistance genes and with the resistance genes *Ht1*, *Ht2*, *Ht3*, and *Htn1*. Samples for qPCR analysis were collected 0, 3, 6 10, and 14 days post inoculation (dpi). The DNA content is presented in ηg DNA/g leaf dry weight. The fourth and fifth leaves from three plants were pooled in one biological replicate. In total, nine plants were harvest per treatment and timepoint (*n* = 3 biological replicates). Data from the first replication experiment are presented in the graph. Lowercase letters indicate significant differences between treatments at 10 dpi. Uppercase letters indicate significant differences between treatments at 14 dpi. Means sharing the same letter were not significantly different following Tukey-adjusted comparisons for data with a log-transformation (*p* ≤ 0.05).

Fungal DNA was detected for both interactions in all inoculated samples at 3, 6, 10, and 14 dpi. DNA content increased over time until the last sampling time points at 10 and 14 dpi. The compatible interaction displayed a higher fungal DNA content after 10 dpi compared to the incompatible interaction at a time point where first symptoms became visible. Moreover, at 14 dpi, B37 presented the highest amount of fungal DNA, followed by the compatible interaction on B37*Ht1*. The compatible interaction of B37*Ht1* (inoculated with race 1) presented a higher DNA content at 10 dpi, due to early symptom expression and higher disease severity (Figure 3), when compared to the other lines. At 14 dpi, high fungal DNA contents were recorded in all compatible interactions, ranging from 700 to 3,100 ηg DNA/g dry weight. In the compatible interaction, B37*Htn1* showed lower fungal DNA-content compared to the other resistant lines, which was in correspondence with disease severity (Figures 3, 4).

### Histological Studies

**FIGURE 5:**
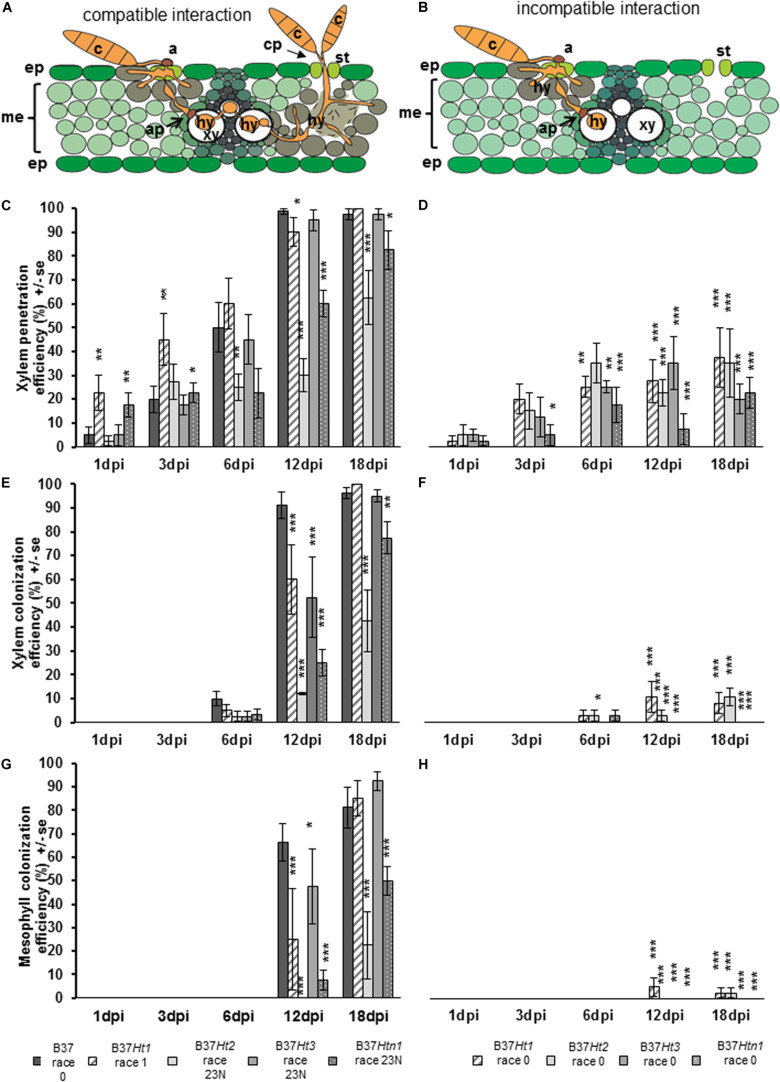
Schematic representation of compatible and incompatible interactions of maize leaves with *Exserohilum turcicum*. Appressorium-like structures are formed from hyphae in the mesophyll with the objective to penetrate into the xylem vessel. The hyphae derive from a germinated spore and penetrate into the epidermis and colonize the mesophyll **(A,B)**. Colonization of the xylem vessel and spread to a new area of the mesophyll with conidiophore formation through the stomata, characterizing a compatible interaction **(A)**. The effect of resistance genes *Ht1, Ht2, Ht3*, and *Htn1* in the respective B37 maize lines on xylem penetration efficiency (XPE), xylem colonization efficiency (XCE), and mesophyll colonization efficiency (MCE) of *E. turcicum* compared between compatible **(A,C,E,G)** and incompatible **(B,D,F,H)** interactions. Four samples were collected 1, 3, 6, 12, and 18 days post inoculation (dpi) per isolate and interaction (*n* = 4). Data represent the 10 penetration sites evaluated per biological replicate. Data from each line with *Ht* resistance genes was compared with B37 by Chi-square test (*p ≤ 0.05, **p ≤ 0.01, and ***p ≤ 0.001). Bars indicate standard error. Symbols indicate: c, conidium; a, appressorium; st, stomata; ep, epidermis; me, mesophyll; xy, xylem vessel; ap, appressorium-like structure; hy, hyphae; cp, conidiophore.

Penetration through the epidermis and into the xylem were observed in both compatible and incompatible interactions (Figure 5A). However, in the compatible interaction, the fungus was able to substantially colonize the xylem tissue resulting in the pathogen hyphae growing through the xylem and into the mesophyll. Mesophyll colonization was primarily observed at greater distance from the penetration site. Under favorable environmental conditions, particularly under high humidity, the pathogen developed reproductive structures (Figure 5A).

Xylem penetration efficiency in the incompatible interactions was between 20 and 40% for the lines bearing resistance genes (Figure 5C). However, in the compatible interaction XPE increased for all lines over time (Figure 5D). The XPE in B37*Ht1* and B37*Ht3* was similar to that of B37. At 3 dpi, XPE was lower in B37*Ht2* and B37*Htn1* than in B37. The XCE was evaluated starting at 6 dpi. For the incompatible interaction, the average XCE was around 10% for B37*Ht1* and B37*Ht2* (Figure 5E). In the compatible interaction, XCE was similar to XPE, since they increase with time after inoculation (Figure 5F).

The MCE was evaluated starting at 12 dpi. The MCE for incompatible and compatible interaction was similar to XCE; all incompatible interactions had less than 5% MCE (Figure 5G). In the compatible interactions, the MCE increased over time. B37*Ht2* and B37*Htn1* presented a lower MCE than B37*Ht1* and B37*Ht3* (Figure 5H). The experiments were repeated and the results obtained in the first run were confirmed by the second run (Supplementary Figures 1–3).

## Discussion

Symptoms of *Exserohilum turcicum* infection in maize leaves had a differential pattern according to the *Ht* resistance gene (Figure 2). In general, chlorosis was associated with resistance, and therefore classified as incompatible interaction, whereas gray-green necrotic lesions were typically observed in compatible interactions. However, lesion size or number of lesions per leaf differed between compatible responses of lines (Abadi et al., 1989). B37, B37*Ht1*, and B37*Ht3* usually had higher disease severity and larger lesions than B37*Ht2* (Figure 3 and Supplementary Figure 1). In the incompatible interaction, the phenotype of infected plants differed according to the resistance gene (Figure 2). The phenotype of plants bearing the *Ht1* gene was always characterized by longer necrotic lesions surrounded by chlorosis (Hilu and Hooker, 1963). Typically, water soaked lesions developed into brownish lesions in the incompatible interaction. Interestingly, the phenotype observed in the incompatible interaction with B37*Ht2* switched between chlorosis and small lesions. The resistance conferred by *Ht2* was characterized by a lower resistance level, as described by Hooker (1977). Conversely, the resistance phenotype expressed conferred by *Ht3* was uniform. Resistant plants always developed chlorotic spots (Hooker, 1981). As described previously, plants bearing the *Htn1* gene switched between lesion-free plants (Gevers, 1975) and plants showing a few wilt-type lesions, similar to small soak spots (Figure 2K). However, plants displaying extended latent period were also observed, as mentioned in the literature (Gevers, 1975).

Molecular studies identified three candidate genes in the *Htn1* locus, which encode wall associated receptor-like kinases (RLKs). These resistance genes produce proteins that are able to recognize pathogen invasion and cell wall disruption (Hurni et al., 2015). Consequently, lower levels of disease severity and fungal colonization in the compatible interaction may be related to this resistance mechanism. The average disease severity on B37*Htn1* was 9.5%, which was low compared to compatible interactions in the other lines (Figure 3). In the qPCR studies, B37*Htn1* inoculated with race 23N (compatible interaction) displayed the lowest fungal DNA content (Figure 4 and Supplementary Figure 2) and XCE and MCE were also delayed, increasing only slowly over time (Figure 5). Therefore, the low fungal DNA content and disease severity observed in the compatible interaction of B37*Htn1* may be related to the resistance mechanism of an extended latent period (Figure 4). The different patterns in hyphal colonization and DNA content confirm that the *Htn1* gene does not offer a completely effective barrier against fungal infection (Gevers, 1975). This supports the molecular analysis that the *Htn1-*gene locus confers a polygenic quantitative resistance against NCLB (Hurni et al., 2015).

Chlorazol Black E (CBE) staining has not been used before to analyze the *in situ* interaction of *E. turcicum* on maize. Regardless of the type of interaction, line, or the staining used to perform the analysis, a stained halo surrounding the infection site was visible at the penetration sites (Hilu and Hooker, 1964). The histological analysis performed with CBE allowed a clear identification of the cell wall. The black color provided by the staining conferred optimum contrast of plant and fungal cell walls for microscopic analysis. An alternative staining technique with calcofluor and distaining with cellulase was previously described for hyphae detection in plant tissue (Trese and Loschke, 1990). However, the hyphal growth could not be clearly observed and, consequently quantified using calcofluor staining. Conversely, CBE staining enabled to visualize and measure XCE and XME, thus differences between interactions could be identified. Before staining, the clearing of specimens was performed by the use of KOH. In contrast to staining with calcofluor (Trese and Loschke, 1990), CBE provides better distinction between xylem and mesophyll tissue due to its affinity for lignified tissue, such as tracheary elements. Moreover, CBE also stained the fungal cell wall, due to its affinity for chitin. CBE has been previously used for staining mycorrhizae (Brundrett et al., 1984). However, CBE staining, as other light microscopy techniques, is not sufficient for a higher level of detail e.g., the identification of cell wall or cell membrane modifications. Therefore, other techniques enabling a higher resolution, such as transmission electron microscopy, are necessary to identify ultrastructural resistance mechanisms like cell wall thickening (Berliner et al., 1969).

*Exserohilum turcicum* is a hemibiotroph characterized by a sequence of biotrophic and necrotrophic phases of infection. The biotrophic phase includes xylem penetration and colonization. The necrotrophic phase starts when the hyphae leave the xylem vessel and provoke plasmolysis of the mesophyll cells until the conidiophores are formed through the stomata (Kotze et al., 2019). Even in the incompatible interaction, the pathogen demonstrated the ability to penetrate into the xylem vessels (Muiru et al., 2008). In the compatible interaction, however, hyphae grew and spread into vascular bundle sheath cells. In all *Ht*-resistant lines tested in this study (*Ht1, Ht2, Ht3*, and *Htn1*), the resistance expressed at the time point of xylem colonization was crucial for further steps in the pathogenesis. Our quantitative analysis of the infection progress suggests that between 3 and 6 dpi is the critical time, during which the resistance mechanism becomes effective to avoid further xylem colonization. Therefore, alterations in the integrity of xylem tissue may be recognized by the host (Bellincampi et al., 2014) between the establishment of infection and the pre-symptomatic state (5–7 dpi).

Differences in symptom expression and fungal colonization, which were observed for each resistant line, strongly suggest that each *Ht* resistance gene encode for distinct resistance mechanisms. In the early infection stages, the *Ht1* resistance displays big necrotic lesions surrounded by chlorosis. However, necrosis observed in the incompatible interaction was not caused by pathogen colonization, since the fungal DNA content was not high. Disease severity was around 50%, but the efficiency of fungal colonization in the mesophyll was low and did not correlate with symptom expression. Therefore, the necrosis observed in B37*Ht1* is considered a strong resistance reaction expressed by *Ht1*. Conversely, *Ht2* represented an unstable phenotype, which was confirmed by lower rates of mesophyll colonization and lower fungal DNA content. The necrosis observed in B37*Ht*2 was caused by fungal colonization. In this case, resistance is expressed by chlorosis or by smaller lesions and a low number of lesions. Instability of the *Ht2* resistance phenotype may be related to the influence of temperature and to the presence of the inhibitor gene *Sht1*. *Sht1* is epistatic to *Ht2* (Ceballos and Gracen, 1989). Therefore, resistance conferred by *Ht2* is considered oligogenic (Hooker, 1977). The *Ht3* phenotype can be easily identified by chlorotic spots. The *Ht3* gene was introgressed from *Tripsacum floridanum* (Hooker, 1981), which is not an alternative host for *E. turcicum*. This implies, that the stability of the *Ht3* resistance phenotype might be related to a mechanism similar to non-host resistance. As an exception, the resistance conferred by *Htn1* is characterized as quantitative resistance (Hurni et al., 2015).

Similar to B37*Htn1*, B37*Ht2* also presented a low average disease severity (17.4%). In the histological studies, XCE and MCE were even lower for B37*Ht2* than B37*Htn1* (Figure 5 and Supplementary Figure 3). However, B37*Ht2* presented a similar fungal DNA content as the other lines. Differences in the average DNA content between the first and the second experiment can be correlated to differences in the level of disease severity (Figures 2G,H), as the race 23N isolate used in these experiments was able to overcome resistance provided by the *Ht2* gene. Even in the compatible interaction, the disease severity was not high which was in agreement with the low XPE, XCE, and XME observed in the histological studies. Therefore, the resistance mechanism underlying *Ht2* may be related to suppression of aggressiveness factors.

*Exserohilum turcicum* produces a non-host specific phytotoxin known as monocerin (Robeson and Strobel, 1982), which may be an aggressiveness factor. In addition to monocerin, a host specific toxin, HT-toxin, has been described to inhibit chlorophyll formation, which might be the main cause of chlorosis and increase on lesion size (Bashan and Levy, 1992; Bashan et al., 1995; Wang et al., 2010; Li et al., 2016). *Ht2* may either encode a mechanism of phytotoxin detoxification (Pedras et al., 2001) and/or synthesis of phytoalexins (Lim et al., 1970), such as a cyclic hydroxamic acid named DIMBOA (2,4-dihydroxy-7-methoxy-1,4-benzoxazin-3-one) (Mace, 1973). Interestingly, some resistance genes appeared to be also effective in reducing disease severity and fungal colonization in the compatible interaction. This supports the hypothesis that R gene related resistance may affect infection also through an underlying mechanisms of quantitative resistance in the maize-*Exserohilum turcicum* pathosystem, as mechanisms of phytotoxin detoxification or the production of phytoalexins may be also related to quantitative resistance (Poland et al., 2009).

From the genetic perspective, *Exserohilum turcicum* interacts with maize following the gene-for-gene concept (Mideros et al., 2018). In such case, each *Ht-*gene should have a corresponding fungal avirulence gene. The first avirulence gene identified for *E. turcicum AVRHt1* corresponds to the *Ht1-*resistance gene (Mideros et al., 2018). *AVRHt1* was expressed *in planta* by a race 23N isolate at 5 and 7 dpi (Hurni et al., 2015), when xylem colonization started (Kotze et al., 2019). Moreover, gene effector candidates encoded a hybrid polyketide synthase:non-ribosomal peptide synthetase (PKS:NRPS) (Wu et al., 2015), and virulence-associated peptidases leupeptin-inhibiting protein 1 fungalysin involved in the biosynthesis of secondary metabolites and cell wall degradation (Human et al., 2020). The increase in XPE and XCE from 3 to 6 dpi indicates that between these time points, the pathogen is releasing virulence effectors. Transcriptional profiles showed that *Ecp6* and *SIX13-like* proteins, similar to the secreted xylem effectors of *Fusarium oxysporum*, were overexpressed at 5 and 7 dpi (Human et al., 2020), which correlates to our findings in the histological studies and indicates that virulence effectors are being released at the time point before symptom expression (5–7 dpi).

The resistance phenotypes expressed by the *Ht* genes are diverse, as the *Ht1* gene expressed necrosis and chlorosis, *Ht2* was characterized by chlorosis and small lesions, *Ht3* showed chlorotic spots and *Htn1* conferred no lesions or wilt-type lesions. These lesions types reflects pathogen colonization, as plants displaying strong necrosis had the complete mesophyll colonized; instead of plants expressing chlorosis, where the xylem and mesophyll were weakly or not colonized. Besides differences on the resistance phenotype, the fungal DNA content was low in the compatible interaction. Indeed, a low fungal DNA content in plants carrying the *Ht* genes even in the compatible interaction shows that these genes have quantitative effect. In fact, *Htn1* was denominated as a source of qualitative resistance. Therefore, *Ht* genes may be associated with or confer additional quantitative resistance.

## Data Availability Statement

The raw data supporting the conclusions of this article will be made available by the authors, without undue reservation.

## Author Contributions

BN, HH, BK, and AT: conceptualization and writing – review and editing. BN, HH, and BK: methodology. BN and HH: software, validation, formal analysis, investigation, resources, data curation, and visualization. BN: writing – original draft preparation. AT: supervision, project administration, and funding acquisition. All authors contributed to the article and approved the submitted version.

## Conflict of Interest

The authors declare that the research was conducted in the absence of any commercial or financial relationships that could be construed as a potential conflict of interest.
