# Supplementary material for: Diversity of Expression Types of Ht Genes Conferring Resistance in Maize to Exserohilum turcicum
Source: Front Plant Sci. 2020 Dec 17;11:607850. doi: 10.3389/fpls.2020.607850 (PMC7773694; doi:10.3389/fpls.2020.607850)
Supplement: Supplementary file 1 [file Data_Sheet_1.pdf]

Supplementary Material

1 Supplementary table

Supplementary table 1. Results from race phenotyping of plants used for *Exserohilum turcicum* DNA quantification and histological studies.

Isolate	Race	B37*Ht1*	B37*Ht2*	B37*Ht3*	B37*Htn1*
172-4	0	A	A	A	A
138-1	1	V	A	A	A
123-3	23N	A	V	V	V

A – avirulent and V - virulent.

2 Supplementary Figures

Supplementary Figure 1. Disease severity and standard error for compatible and incompatible interactions between *Exserohilum turcicum* and the maize lines B37 without *Ht* resistance genes and those with resistance genes *Ht1*, *Ht2*, *Ht3*, and *Htn1*. Disease severity was evaluated 14 days post inoculation (dpi). The graph presents data from the second replication. Data from each line with *Ht* resistance genes was compared with B37 by Mann-Whitney-U test (* $p \leq 0.05$, ** $p \leq 0.01$, *** $p \leq 0.001$).

Supplementary figure 2. DNA-contents with standard errors for compatible and incompatible interactions between *Exserohilum turcicum* and the maize lines B37 without *Ht* resistance genes and those with resistance genes *Ht1*, *Ht2*, *Ht3*, and *Htn1*. Samples for qPCR-analysis were collected 0, 3, 6, 10 and 14 days post inoculation (dpi). The DNA content is presented in ηg DNA / g leaf dry weight. Only data from the second replication experiment are shown in the graph. Lowercase letters indicate significant differences between treatments at 10 dpi. Uppercase letters indicate significant differences between treatments at 14 dpi. Means sharing the same letter were not significantly different following Tukey-adjusted comparisons for data with a log-transformation ($p \leq 0.05$).

Supplementary figure 3. Data from the second repetition for the effect of resistance genes *Ht1*, *Ht2*, *Ht3*, and *Htn1* in the maize line B37 on xylem penetration efficiency (XPE), xylem colonization efficiency (XCE), and mesophyll colonization efficiency (MCE) of *Exserohilum turcicum* compared between compatible (A,C,E) and incompatible (B,D,F) interactions. Samples were collected 1, 3, 6, 12, and 18 days after inoculation (dpi) with one isolate for each interaction. Data is from 10 penetration sites in four leaf segments (n=40). Data from each line with *Ht* resistance genes was compared with B37 by Chi-square test (* $p \leq 0.05$, ** $p \leq 0.01$, *** $p \leq 0.001$). Bars indicate the standard error.
