# Supplementary figures and images for: Diversity of Expression Types of Ht Genes Conferring Resistance in Maize to Exserohilum turcicum
Source: Front Plant Sci. 2020 Dec 17;11:607850. doi: 10.3389/fpls.2020.607850 (PMC7773694; doi:10.3389/fpls.2020.607850)

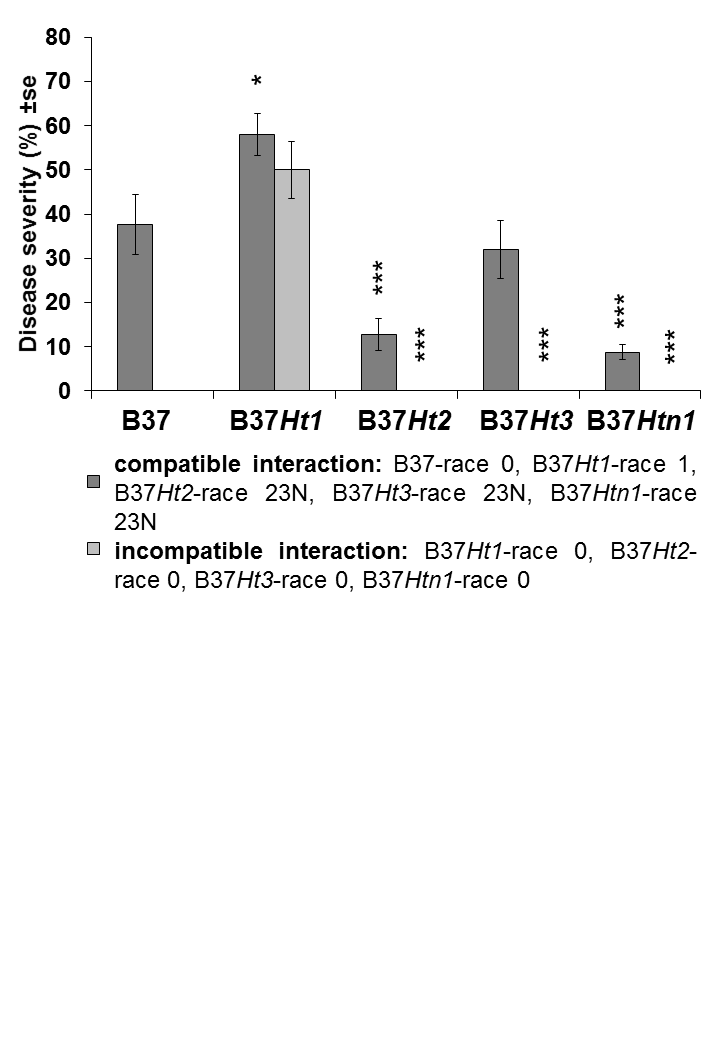

Supplement: Supplementary file 2 [file Image_1.TIF]

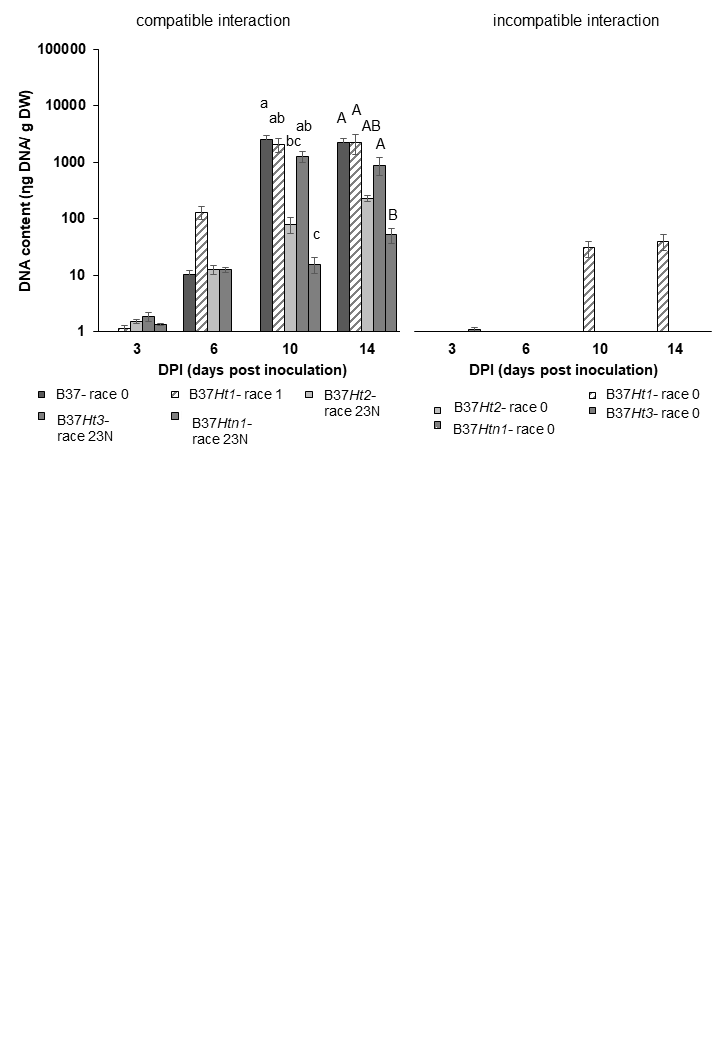

Supplement: Supplementary file 3 [file Image_2.tif]

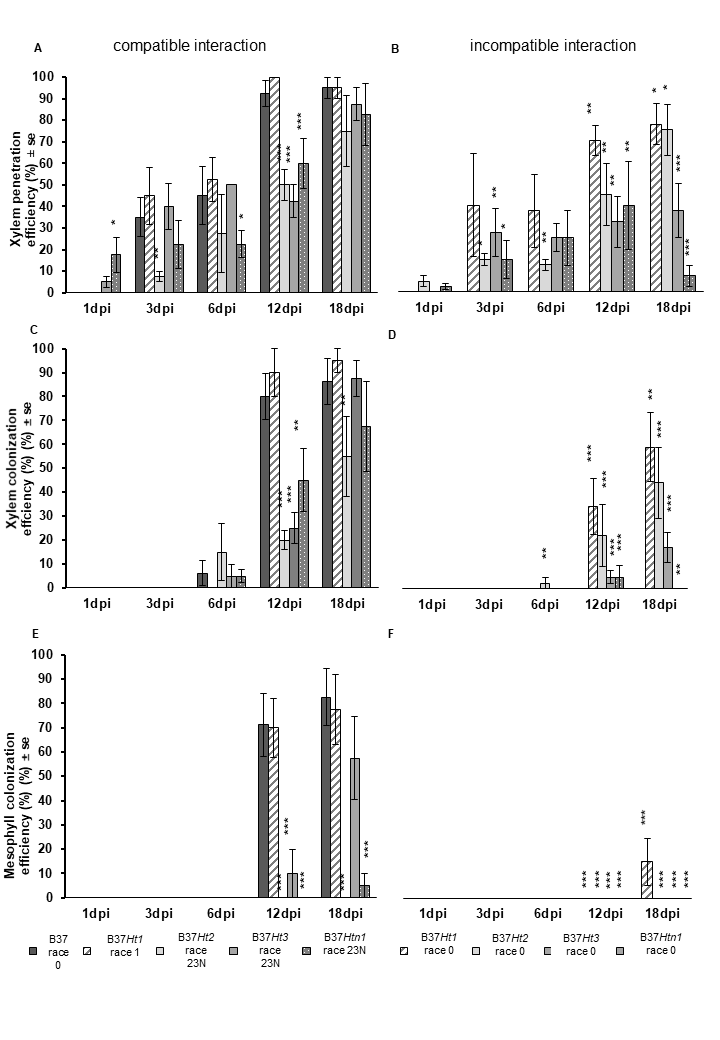

Supplement: Supplementary file 4 [file Image_3.tif]
